# Agri-Food Value Chain Traceability Using Blockchain Technology: Portuguese Hams’ Production Scenario

**DOI:** 10.3390/foods12234246

**Published:** 2023-11-24

**Authors:** Miguel Arvana, Andre Dionisio Rocha, Jose Barata

**Affiliations:** NOVA School of Science and Technology, Center of Technology and Systems (UNINOVA-CTS) and Associated Lab of Intelligent Systems (LASI), NOVA University Lisbon, 2829-516 Lisbon, Portugal; m.arvana@uninova.pt (M.A.); jab@uninova.pt (J.B.)

**Keywords:** agri-food, value chain, blockchain technology, traceability, digitization

## Abstract

The globalization of food markets has led companies to buy products not only locally, but also from other corners of the world. This has introduced complexity into supply chains, as products have to move longer distances and pass through more companies before reaching the end consumer. The meat industry has been no different. Events such as animal disease outbreaks have diminished consumer confidence in the industry and the supply chain. Coupled with this, consumers started demanding “more transparent” meat products. This has led companies to think about new traceability systems, which continue to enforce food safety and health rules, but at the same time enhance and make transparent to the consumer the origin and constitution of their products. This article proposes a traceability system in the agri-food (meat industry) with a multi-chain architecture, among them, blockchain. The use of blockchain in the traceability system helped to mitigate the omission of relevant data for the traceability process, allowing us to guarantee the immutability, reliability, and transparency of the data along the value chain. At the same time, the system was able to reduce the time of the traceability process by giving the user the possibility to access the traced information via a unique product identifier.

## 1. Introduction

In the 21st century, eating habits have changed dramatically, as consumers are more informed than ever and more demanding about their food. Of all foods, meat and meat products have been one of the most under pressure from consumers. The demand for “more transparent” products has led companies to seek and develop new methods to value and disclose to the consumer the origin and constitution of their products [1]. However, current traceability systems, which operate in a rigid, unilateral way and in which the entity in possession has all the information, have been developed only with the aim of satisfying quality and food safety requirements.

Industry 4.0 (I4.0) aims to simplify, automate, and connect a large part of the production stages, using new storage methods, device connection, and information sharing [2]. Companies now have a set of solutions that can take their traceability systems further, allowing them to value their products with the addition of more information, which in turn will open the door to a new concept of traceability [2,3].

End-to-end traceability presupposes the inclusion of all stakeholders along the entire value chain, to value and make transparent to the consumer the origin and processes through which the product has passed [4,5]. Applying blockchain to traceability systems will facilitate the acquisition and sharing of data. It can provide a global view of the supply chain, allowing all stakeholders to track the movement of products from producer to consumer. At the same time, blockchain also increases transparency, which helps consumers regain trust in the supply chain [6], of which it was once lost due to animal disease outbreak events.

To trace a meat product (meat industry) from its production to the consumer, with transparency towards the consumer being the key point, the following question can be asked:

RQ: How can a cloud computing system be designed that increases transparency towards the consumer about an agro-industry value chain?

The hypothesis proposes the design of a multi-layered solution, namely blockchain, which makes it possible to extract and store all the data necessary for product traceability throughout the value chain, ensuring its reliability and immutability.

This paper is structured as follows. Section 2 defines the literature review, including studies on the agri-food sector, meat industry, traceability in the agri-food sector, and blockchain in the agri-food sector. In Section 3, the real application scenario for developing the solution is presented. Section 4 describes the whole structure of the multilayer architecture. In Section 5, the architecture described in the previous section is implemented, using the Hyperledger Fabric (HLF) framework to implement the blockchain layer. An application was also developed for user interaction with the system. Section 6 focuses on the results of the practical use of the developed application. Finally, in Section 7, an analysis of the work developed and its limitations in the scope of the use of blockchain applied to traceability systems is made. Finally, this chapter also addresses the way forward for future research.

## 2. Literature Review

In this section, a literature review was carried out covering topics such as the agri-food sector, the meat and livestock industry, traceability in agri-food, and blockchain in agri-food.

### 2.1. Agri-Food Sector

Food is and always will be a central need for all societies. The agri-food sector covers all activities related to the processing of raw materials and foodstuffs and their distribution [7]. Naturally, a sector such as agri-food is subject to constant change, as modern agri-food supply chains have moved from autonomous and independent local actors to globally interconnected systems of multiple actors linked by complex relationships [8]. Recent events such as Brexit, the SARS-COV-2 pandemic, and the Russia–Ukraine conflict are examples that show the complexity and interconnectedness of agri-food systems [2,9].

However, changes in the sector are also influenced by the needs of consumers. Consumers have been faced with frequent incidents of fraudulent practices, which have exposed the lack of transparency in agri-food supply chains that prevents them from making informed choices. The growing awareness of healthy habits and the consequent demand for “more transparent” products from safe sources have been highly recognized by all stakeholders in the supply chain [1,10].

Initiatives such as “Farm to Fork”, developed by the European Commission, aim to build a supply chain that works for the consumer, the producer, the climate, and the environment, but at the same time recognizes the importance that consumers attach to traceability systems. This requires the development of mechanisms that use traceability data to fight food fraud [11].

#### Meat and Livestock Industry

Population growth and rising wages have translated into widespread growth in the agri-food sector, making it dynamic overall. At the same time, the sector’s growth has substantially affected how meat and meat products are perceived by consumers. Going back a little in time, we can obtain a better sense of why this shift has occurred.

Historically, the increase in livestock production and the demand for meat products were supported by combinations of conventional techniques and cross-breeding [12]. Today, conventional techniques are not sufficient to keep up with market needs. This puts pressure on producers to reformulate their production methods. One of the paths followed by producers was the use of growth promoters and antibiotics. At the same time, consumers have become more demanding in factors such as animal welfare and health, reduced use of antibiotics, and more transparency of the provenance of products [13], starting to opt for the consumption of organic and more sustainable meats [14]. This led the European Union in 2006 to ban the use of antibiotics, hormones, and growth promoters. However, not all countries have followed suit, as certain hormones increase feed retention and, in turn, livestock productivity [12].

At the same time, the meat sector has also grown. Eating habits have changed and there has been a greater demand for prepared and processed products. This has led to changes in working methods and in the way industries operate [15]. Associated with the rapid growth of sectors and markets were outbreaks of animal diseases such as African swine fever, avian influenza, and bovine spongiform encephalopathy (BSE), or the presence of chemicals in feed [16,17]. These issues have become alarming for the consumer as they affect the quality and safety of products on the market. This led to the need to implement traceability systems, capable of tracing from “farm to fork”, in order to restore and increase consumer confidence in food safety [16]. As you can see, consumers have the power to influence meat production systems by their increasing demands. Issues such as food safety, food quality, and transparency of product provenance have gained much attention due to the growing interest of consumers in the origin of products [18].

### 2.2. Traceability in Agri-Food

These days, the distance that food travels from the producer to the final consumer has increased due to globalization. However, maintaining food quality and safety throughout the supply chain has become challenging. In recent decades, the credibility of the food industry, more specifically the meat industry, has been challenged due to numerous crises such as those mentioned in the previous point. In response to the growing questions, numerous laws, policies, and standards relating to food safety and quality have been developed and improved. Traceability attempts to satisfy food safety and quality requirements. Various standards define it (EU Regulation (EC) No 178/2002 [19], ISO 9000:2015 [20], FAO CODEX Alimentarius CXG 60-2006 [21]) as the ability to trace the movement of a specific food through all stages of production, processing, and distribution [22].

However, consumers continue to demand to know more information about the origin of products and their ingredients. Thus, to receive quality and safe food, there is a need to build and develop traceability systems. However, many companies use their traceability systems to comply with regulatory requirements, which in the case of Portugal are defined in EU Regulation (EC) No 178/2002. Companies are obliged to track their products internally and externally [19], only for that reason and not to conquer or please consumers.

**Figure 1:**
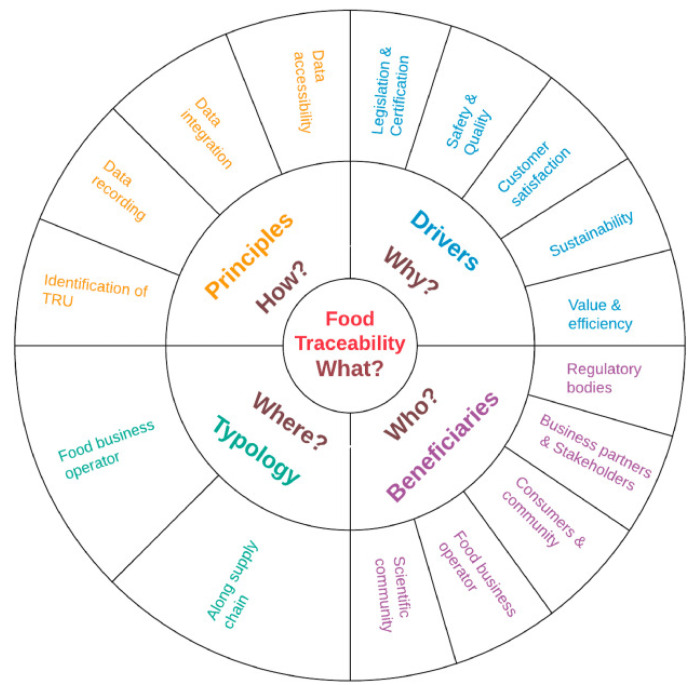
Theoretical representation of food traceability [24].

However, the transparency of the products’ origin has influenced traceability technology’s evolution. According to Sunny, J et al., traceability and transparency are two terms that have been confused in the supply chain context because, despite being interconnected, they assume different meanings [23]. Traceability, as mentioned earlier, is the ability to access information about anything part of the supply chain. That is, what, how, where, why, and who, as shown in Figure 1. On the other hand, supply chain transparency is the ability to access information related to the product and its processes throughout the supply chain. In short, traceability allows for transparency in the supply chain.

#### 2.2.1. Types of Traceability

According to [24], different authors classified food traceability in many ways. Some authors classify traceability as downstream and upstream traceability, which defines the movement of the product along the supply chain. Other authors divide food traceability into six groups:

- Product traceability;
- Process traceability;
- Genetic traceability;
- Traceability of entries;
- Traceability of diseases and pests;
- Traceability of measurements.

However, other authors divided it into two groups: logistical traceability and quality traceability.

There are different points of view on how to classify food traceability. The definition present in the literature such as [4,19] indicates that any food traceability system must be able to manage any information related to the product. Consequently, authors classify traceability as internal traceability and external traceability.

Internal traceability concerns the recording of all data and processes related to the product’s processing within a company, such as the connection of a new product to its original raw materials. This link is made via the lot number of the traceable item. This principle applies even when the traceable item is part of a hierarchy, such as pallets or containers [24,25].

External traceability requires that all items produced in a company are uniquely identified. For external traceability to be maintained, the identification numbers of traceable items must be shared among all actors in the distribution chain through product labels or documents. External traceability allows upstream traceability (to the supplier) or downstream traceability (to the customer) [24,25].

However, entities such as the GS1 Global Traceability Standard and the Codex Alimentarius Commission propose end-to-end traceability, a new approach. Looking at the supply chain more closely, we conclude that each company and organization is responsible for its traceability data. In order to achieve end-to-end traceability, it will be necessary to access and combine the data from the multiple entities involved in the product life cycle. This new approach will bring the possibility of tracking and locating an object throughout its entire life cycle through all parties involved in its production, both upstream and downstream. To fulfill the goal of end-to-end traceability in the supply chain, all companies and operators in the chain must use a set of standards so that their systems and technologies used, even if different, can be integrated and communicated [4,5].

#### 2.2.2. Identification and Traceability Tools

The supply chain area has undergone significant technological growth due to the need to provide safe food and tools that mitigate events that jeopardize food security. To this end, the application of these technologies has been focused on traceability systems. Most current traceability systems are centralized, which can raise trust issues such as tampering and falsifying information. Furthermore, centralized systems are vulnerable, as a single point of failure can lead to the entire system crashing. However, technological advances have provided technologies such as blockchain, which allow traceability systems to be decentralized, making them more reliable and less prone to fraud and information tampering [26]. In traceability systems, data collection is very time-consuming when performed manually, as the worker must record the information at each point of the activity or in each process applied to the product and subsequently enter the data into the computer system. This manual process can lead to errors in collecting information or entering the data. In order to mitigate human errors and, in turn, make traceability processes along the supply chain more efficient and accurate, identification technologies are used for products and information capture [27] such as Barcode, Radio-Frequency IDentification (RFID), Near Field Communication (NFC), or Wireless Sensor Network (WSN) [28].

Thus, the work presented proposes a technological ecosystem capable of tracking along the agri-food value chain. In order to guarantee the integrity of this traceability, a solution that uses blockchain technology is presented. In this way, it is possible to ensure that the final consumer can audit a particular product through the data stored in the blockchain. This will include data such as the parties involved in the process, the date on which each process was carried out, and other critical data. A case study is then presented where the work carried out was tested and demonstrated. This case study was applied to the meat industry, but specifically to the production of hams.

### 2.3. Blockchain in Agri-Food

Today, blockchain applications go far beyond digital currencies, extending from financial sectors, healthcare, supply chain management, and market monitoring to copyright [29]. With the growing popularity of the internet and the introduction of online digital platforms for online business transactions and collaboration, a new business dynamism has emerged, increasing trust and transparency between trading partners. The introduction of blockchain on digital platforms has offered new value propositions, from the decentralization of data storage to the decentralization of management and decision making [30].

Although agri-food supply chains today are globally interconnected, all information associated with safety, sustainability, origin, and many others are either expressed on paper or stored in centralized databases that only enforcement authorities can access, making the agri-food sector one of the least digitized sectors [31].

The use of blockchain technology in agri-food can change the way the sector behaves in various situations. The application of smart contracts in the supply chain can play the role of regulatory bodies, automating the processing and certification of transactions between elements of the chain. In other words, the blockchain must work in parallel with the current certifications and standards used in each supply chain. According to [32], blockchain is used to store the data collected by sensors in the field, as it makes it impossible to change the history of the data. These data is then used for the certification process of organic farming, increasing consumer confidence in the control mechanisms of organic production. Another approach is to integrate blockchain into current certification systems. According to the authors in [33], they propose using blockchain tokens and smart contracts as reliable certification tools (third-party certification). This is to provide stakeholders in the value chain with a fraud-resistant tool.

Blockchain offers decentralized and immutable record storage, as well as access to the data beyond the nodes of the network. This serves as a tool to increase trust between agents in the chain, thanks to the ease of record validation. Finally, the use of blockchain can improve the traceability of products throughout the supply chain, getting closer to achieving the concept of “farm-to-fork” [31].

However, doubts remain as to whether it is feasible to apply blockchain-based traceability services in the agri-food supply chain. Growing consumer awareness and the tightening of food safety policies have made the notion of traceability increasingly important. Analyses of agri-food supply chains indicated that traceability can improve food safety as well as bring benefits to their sustainability. At the same time, from a technological perspective, the traceability services currently used, such as RFID, NFC, bar code, etc.,present deficiencies in the security, transparency, and credibility of the information. Given this, some authors have studied how the agri-food supply chain can use blockchain to its advantage. They ended up concluding that blockchain can improve the traceability of produced products, guaranteeing the credibility and transparency of the origin of the data [34].

Blockchain technology is already being applied in real cases of traceability. Some of these are Pork Meat Traceability (TE-FOOD), Celeia Dairy (OriginTrail), Wine Blockchain (EzLab), Bytable’s (Trace My Egg), and BeefLedger, but the most famous case of blockchain technology application was developed by Walmart together with IBM, for the traceability of mangoes [31,35]. In addition to the above-mentioned cases, many others have been evaluated, concluding that the meat supply chain can be ideal for blockchain application projects. A chain of this type involves several actors at different stages, where it is linear and there is a genuine interest on the part of the consumer for traceability, which is mostly related to the origin of the meat [35].

Although most blockchain applications are product-focused, there are also blockchain-based applications that lead to sustainable business models. This is because agri-food businesses have been asked to align themselves with the growing demands of social responsibility and sustainability. In other words, the focus shifts from product traceability to production traceability [36]. Several authors, such as Kamble et al., have pointed out that the use of blockchain can change the way transactions are carried out, reducing the number of intermediaries in the supply chain [37]. In addition, the use of blockchain technology from a production traceability perspective can also help ensure the long-term viability of environmentally friendly supply chains [36].

## 3. Application Scenario

The proposed study will use a real application scenario to develop a solution that meets the real needs of a traceability system in the meat industry. In this scenario, the products to be tracked are black pork hams from Alentejo, produced in this Portuguese region known for its quality charcuterie. The company that provided this case study intends to use traceable information to control the quality of its products and enhance them and make the product’s origin transparent to the final consumer.

**Figure 2:**
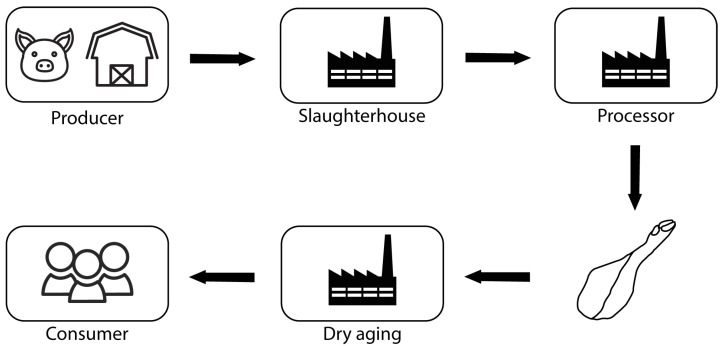
Product life cycle.

The product’s life cycle (Figure 2) begins with the production of animals (pigs). The producer is responsible for their feeding and welfare. He sells the animals to a processing company and they are transported to the slaughterhouse, where they are slaughtered and, in turn, stored until the processing company collects them.

The carcasses are collected and processed, resulting in different pieces of meat, one of which is the leg that gives rise to the ham. The ham is created on the premises of the processing company and is given a unique identification number. This goes on to dry aging at another company, where it remains for 1 to 3 years. After the dry aging period, the ham is then ready for sale.

Once the ham is in the consumer’s possession, if they want to determine the origin or conditions of the product throughout its life cycle, they will have to consult the seller, who will not have all the information available or will rely on the information that comes with the product at the time of purchase. This information may be falsified, have errors, or even be superficial, omitting transport, storage, and animal welfare conditions. Even if some of these data is entered into a shared centralized database, problems such as tampering or deletion and hacking of the system can occur. Most traceability systems are limited to the operations of the owning company, leading them not to share their platforms due to a lack of trust or competitiveness in the industry.

### System Objectives and Requirements

**Figure 3:**
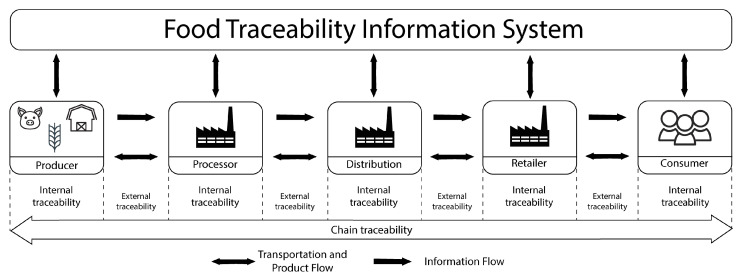
Theoretical representation of food traceability. Adapted from [38].

Based on the traceability requirements for the food chain and the problems that the application scenario presents, a conceptual framework was developed for an end-to-end traceability system in the food industry, which can be represented by Figure 3.

In this framework, it is considered that all actors are part of the supply chain, being producers, meat processors, stockists, resellers, and consumers. It is also considered that all these actors have internal (inside the company) and external (outside the company) traceability.

To achieve traceability throughout the chain, the application scenario described previously in this section is considered, as well as the conceptual framework of the food traceability system. Thus, the objectives for the end-to-end traceability system can be defined as the following:

- Provide the customer with the origin of the product, as well all the processes to which it was submitted.
- Ensure the reliability and immutability of the data.
- Mitigate the omission of relevant data for the traceability process, as well for product transparency.
- Allow the consumer to consult the information traced from the unique identifier associated with the ham.

In addition to the objectives mentioned above, it is necessary to take into account that users must interact with the traceability system and also how the data collected from each process will be stored. Then the system requirements can be described by the following topics:

- Since the system is based on the end-to-end traceability concept, it will have to cover all the processes performed on the product. As such, it will have to be horizontal to the product lifecycle, requiring a common platform to be provided to all stakeholders.
- To minimize data tampering and all other problems associated with a centralized system, a decentralized storage system should be used, based on a solution that ensures data reliability and immutability.
- To mitigate the omission of data and make the origin of the product and all the processes to which it was submitted to the customer, a logic of transactions and processes must be applied to the product’s life cycle.
- To increase confidence in the data and its origin, only authorized and registered users will have access to the platform and its features.
- To facilitate the collection of traceable information and its subsequent sharing with the consumer, the system will have to support the storage of different data models.

## 4. System Architecture

**Figure 4:**
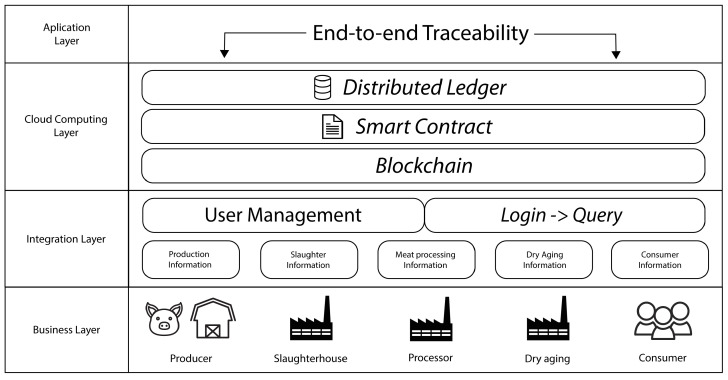
Proposed architecture.

A multilayer architecture is proposed to meet the scenario presented above, as well as to meet the objectives and requirements of the system. As shown in Figure 4, this architecture consists of four layers:

- Business Layer: The entire business process of the supply chain associated with the scenario is described in this layer. Thus, it is observed that the supply chain consists of five types of organizations: the producer, who raises the pigs; the slaughterhouse, which performs the slaughter; the processor, who is responsible for cutting the pig’s carcass and creating the ham; the dry aging, which deals with the process of dry aging the ham; and finally, the consumer, who buys the ham;
- Integration Layer: Perhaps the most critical layer of the entire architecture is responsible for integrating the different technologies that will be part of the final system. This layer includes a set of modules for collecting and sharing information. These are horizontal to the business layer, fulfilling the requirement of a common platform for all parties involved in the product life cycle. This layer also includes a module for managing users and one for controlling access (login) to the platform. These modules are directly related since each type of user will have different access permissions to different features. These enforce the necessity of increasing confidence in the origin of the data;
- Cloud Computing Layer: It is in this layer that a blockchain network is instantiated. Its features are ideal for meeting system requirements. Blockchain is a Distributed Ledger Technology (DLT), which means that the ledger (set of stored data) is distributed across multiple devices at different geographic points. In turn, the data is stored in the form of a ledger. This feature of blockchain technology enforces the necessity of data tampering and mitigates the problems associated with a centralized storage system. Still, in this layer, associated with the blockchain network, is the smart contract module. Thanks to its ability to create events and actions according to a real case, a transaction and process logic that describes the product life cycle can be defined. At the same time, the smart contract can include state variables, functions, and structures, making it possible to store different data models. The use of smart contracts makes it possible to comply with the need to store different data models and the requirement to mitigate the data omission, making the product’s origin and all the processes to which the product was submitted appear;
- Application Layer: The organizations involved in the supply chain will interact with the system through this layer, being able to manage all information and transactions. Through the product’s unique identifier, consumers can access the product’s tracked information.

The proposed architecture for the system covers the entire food supply chain, including unreferenced entities. Therefore, organizations that participate in the supply chain can carry out their transactions without any problem, knowing that all the information necessary for the traceability of the final product is being stored securely and transparently. This model allows for faster product search, quality checks, and more efficient collection of products in case of danger to public health. In turn, customers have a set of information that shows the product. As a result, organizations can gain more trust from their customers.

### 4.1. Data Model

When the consumer wants to query the product information using the system based on the proposed architecture, there must be records of all the processes that product has undergone. For the presented scenario, the information to be collected goes beyond the change in owner or state since end-to-end traceability must include external and internal traceable information in order to be complete. In the case of the proposed work, the product undergoes a transformation during its life cycle, going from pork to ham. However, the ham, or leg, is not the only piece from the pork processing process, so it becomes necessary to create a middle ground between the pork and the ham, which is capable of recording all the pieces. This implies that a record is created at the time of the processing.

For this, three types of records are considered. One refers to the external traceability of the herd. The other refers to the internal traceability of the pig’s herd in the processing, and finally, one refers to the ham.

**Figure 5:**
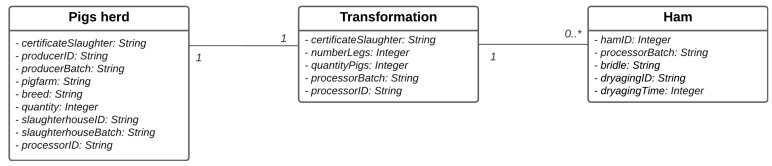
Logical data model.

For the traceability process to be possible, it is necessary to relate the different records. Figure 5 represents the simplified data model that is used to describe the data. However, the database will not be a replica of the model, as the blockchain will manage the data. The data model is made up of three entities: Pigs herd, Transformation, and Ham. The relation between the Pigs herd entities and Transformation is 1 to 1. The relation between the Transformation and Ham entities is 1 to 0..*.

#### Users

To increase confidence in the data and its origin, only authorized and registered users will have access to the platform and its features. These features differ depending on the type of user.

A user can be described as follows:

- ID: User’s unique identifier.
- password: Password for login.
- type: User type (admin, producer, slaughterhouse, processor, or dry aging).

In order to ensure that the tracking process and subsequent collection of information have some level of reliability and security, the proposed blockchain-based system must be permissioned, that is, only users with permission can interact with the system and execute smart contracts. Table 1 shows in more detail the permissions for each type of user.

- Admin: This user is responsible for managing the platform and its users. It is considered the maximum user and is allowed to execute all smart contracts.
- Producer: These types of users are animal producers. In order for the traceability process to be correct, the origin of the product must always be registered first; therefore, it is the first user to appear in the supply chain, being the only one who can create new records of the “Pigs herd” type.
- Slaughterhouse: These types of users are responsible for slaughtering the animals from the producer. It can update the state of “Pigs herd”-type records.
- Processor: These types of users are responsible for transforming the raw materials. The transformation process for this scenario is the processing of meat from the pig carcass, allowing for the creation of new products, among which is the leg for ham. With this, users of this type have permission to change the state of “Pigs herd” records and create new “Ham” records.
- Dry aging: These types of users are responsible for carrying out the dry aging process, which can last between 1 to 3 years. They can only change the status and information of “Ham”-type records.
- Consumer: Represents the last user to intervene in the product’s life cycle. The consumer uses the unique identifier of the product to find out about the origin of the raw material and all the processes which the product passed through during the production cycle.

### 4.2. Transaction Logic

Transaction logic defines the behavior of the system for the phenomenon of state changes along the production chain.

Knowing in advance the application scenario, the data model, the participating entities, and their permissions associated with traceability in the production chain, we can build a model that defines the system’s transaction logic.

The starting element for building the model is the type of bearer of the product since each one will have different information to share with the system. To define the stages of production, it is necessary to frame the stages through which the product has passed in the production chain with each of the actors involved in the product production. Going down another level in the model, it is necessary to define when (production stage) and which product bearer will be able to update or create new records. The data model gives us information on how to define this part of the model.

**Figure 6:**
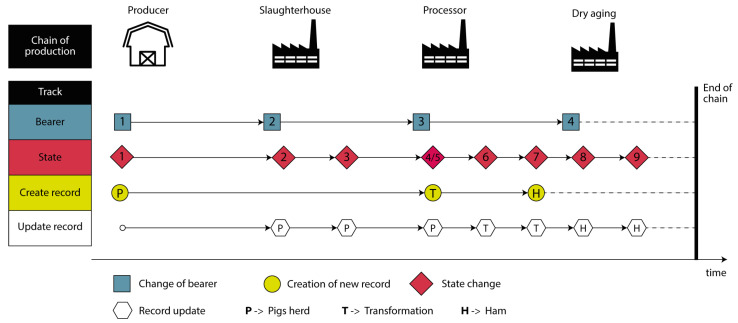
Transaction model.

Analyzing all the variables mentioned above, we now have a transaction model like the one shown in Figure 6.

Five bearers of the product are considered, with the last being the consumer, who will not influence the transactions. Nine states are also considered. These represent the different processes that the product goes through. The states are distributed, depending on the user, as follows:

- One state for producer (state 1);
- Two states for the slaughterhouse (states 2 and 3);
- Four states for the processor (states 4, 5, 6, and 7);
- Two states for dry aging (states 8 and 9).

States four and five correspond to the same moment in time, but are associated with different records.

Two types of actions have been defined: “Create record” and “Update record”. Transaction model actions are associated with permissions by user type (Table 1) as follows:

- “Create record” action -> “Create record” permission;
- “Update record” action -> “Update information” permission.

The “Create record” action creates a new record of type “Pigs herd” , “Transformation”, or “Ham”. The type of record created depends on the type of user. The “Update record” action updates the information of records (pigs herd, transformation, or ham) that already exist. Once again, the type of record to be updated will depend on the type of user and the moment in time.

Considering the type of information that each type of user has to provide, the distribution of states by the type of user, and the application scenario, it becomes possible to define the actions that must be carried out in each state. State 1 corresponds to the creation of a new record of type “Pigs herd” by the user of the producer type. This state represents the beginning of the traceability process as well as the product life cycle. States 2 and 3 are performed by slaughterhouse-type users and correspond respectively to the reception of animals at the slaughterhouse and to slaughter. In any of these states, the “Pigs herd”-type record is updated. State 4 is performed by users of the processor type and corresponds to the arrival of animals at the meat processing company. In this, the “Pigs herd”-type record is updated. At the same time, state 5 occurs and, similarly to 4, it is executed by users of the processor type. This marks the beginning of the process within the transforming entity. This corresponds to the creation of a new record of type “Transformation”. State 6 is performed by users of the processor type and corresponds to the cutting of animals. In this, the “Transformation”-type record is updated. State 7 is executed by users of the processor type and corresponds to the creation of a new ham. In this one, a new record of type “Ham” is created and the record of type “Transformation”, corresponding to the ham, is updated. States 8 and 9 are performed by dry aging users and correspond respectively to the reception of the ham in the dry aging process and the completion of the dry aging process. State 9 indicates the end of the traceability process, as well as the end of the production chain.

## 5. Implementation and Tests

**Figure 7:**
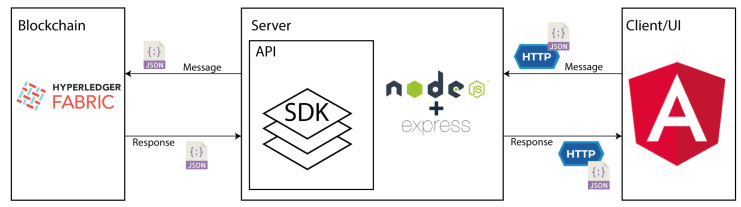
Implementation model with technologies.

Like any other technological solution, it was necessary to create an implementation model with the respective technologies to be used. This is represented in Figure 7. In order for the traceability system to be applied to the scenario in question, users need an interface that allows them to enter information relevant to the traceability process. A simple web application was then developed that can individualize each user. Each one has access to the smart contract functionalities and can enter and consult the relevant data.

For the development of the web application, the following technologies were used:

- Node.js: It translates into an open-source, asynchronous, and event-driven environment, which executes JavaScript code server-side, allowing for the development of web servers with multiple functionalities [39].
- Express: Express is a Node.js-based framework used to build web applications. Like Node, it is open source and provides a robust set of tools for HTTP servers [40].
- Angular: Angular is a TypeScript-based web framework that allows you to create single-page applications [41].

For the implementation of the blockchain, the Hyperledger Fabric (HLF) framework was used. The choice of this framework took into account the following considerations:

- HLF is an open-source platform developed under the umbrella of the Linux Foundation. The latter aims to develop projects aimed at promoting transparency and the fight against corruption. It was designed to be used in business environments, as it has a highly modular and configurable architecture, allowing for its application in numerous fields such as industry, banking, insurance, healthcare, and supply chain [42];
- Supports a range of different smart contracts programming languages such as Java, Go, and Node.js [42];
- The HLF platform is permissioned, which means that all blockchain participants are known and identified [42];
- HLF provides a Software Development Kit (SDK) in several programming languages. It supports building web applications on top of the blockchain network. At the same time, it allows developers to connect applications to network nodes, perform smart contract functions, and access the data in a more user-friendly way [42].

### 5.1. Blockchain Network

For the construction of the blockchain network, some factors about the application scenario were taken into account. As mentioned before, many companies in the meat sector have their traceability systems in place simply to comply with regulations and not to take advantage of the information made available to them. In this case, although we have four different types of entities that will intervene in the production of ham, the initiative for this system came from only one, which is the processor type, since it is the one that intends to add value to its products.

Furthermore, it is necessary to take into account the conditions of producers in Portugal. There is a very small number of animal productions that have a computer infrastructure capable of supporting a node of a blockchain network. In addition, many of these productions are small family productions and do not intend to evolve to improve their IT infrastructures. The same situation happens with slaughterhouses and dry aging companies, which, despite having the possibility of hosting a node, are not interested in bearing the costs of managing one, and in the end, will not benefit directly from the traceability of the final product.

**Figure 8:**
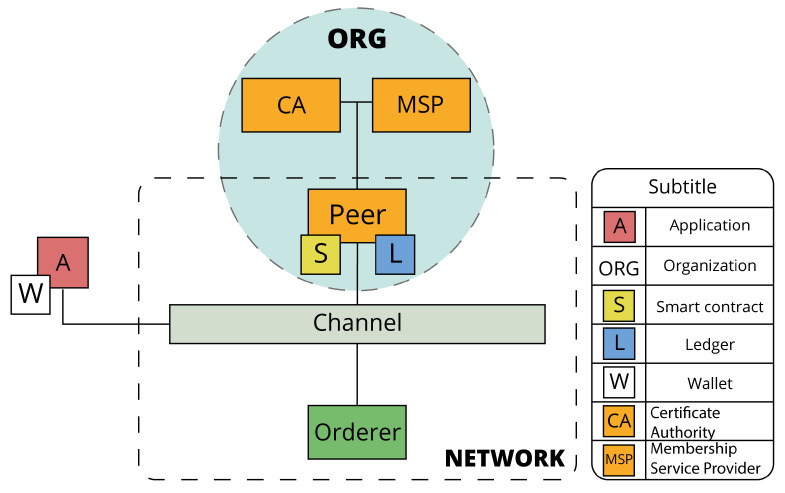
Network architecture.

Taking into account the above-mentioned points, the architecture of the blockchain network can be represented by Figure 8.

It was then decided that the network should consist of an organization with a peer node, a channel, an application, and an orderer node. The management of the system will fall to the company that provided the case study, which in this case is of the “processor” type. This is represented in the blockchain network by the organization and by the peer node.

The network architecture may seem simple, but given the modularity of the HLF architecture, as new companies gain interest in digitizing traceability, new organizations, peers, channels, and ordering nodes can be added. As the network grows, its administration will be distributed among all participating organizations.

### 5.2. Smart Contract

Smart contracts are a fundamental part of any blockchain-based solution. This section explains the implementation of the smart contract that allows for ensuring the storage of different types of data and mitigating their omission and, at the same time, making the origin of the product and all the processes to which it is submitted appear.

The smart contract defines the rules and transaction logic that control the product lifecycle, allowing access to the entire history of transaction records as well as the current status of the product.

To ensure that only authorized entities can update the data relevant to their work, the smart contract is designed and programmed in such a way that it only allows transactions to be approved if they are sent by authorized entities for that state. In addition, the smart contract is also designed and programmed so that it validates the data entered by each entity in each state of the product’s life cycle. It enforces the logical data model (Figure 5) in accordance with the transaction model (Figure 6).

As indicated in Table 1, entities can update the status and product information; however, only when they are the product bearers. To define the transaction logic that controls the product’s life cycle, it was necessary to create the nine states for the product, which are associated with the entity’s possession of it. In Table 2, you can see the nine states and the product bearer for each of them.

Associated with the change in status is the update of information and/or the creation of a new record. As can be seen from Table 3, only in states “HERD_CREATED: 1”, “HERD_IN_PROCESSING: 5”, and “HAM_CREATED: 7”, new records are created; in the others, only updates of existing records are made.

Having said this, and taking into account the permissions of each user type, actions per state, product carrier per state, the data model, and the transaction model, we developed a set of functions that allowed us to create a logic of transactions that represents the product’s life cycle. In Table 4, all the functions that are part of the smart contract developed in this work are described.

**Figure 9:**
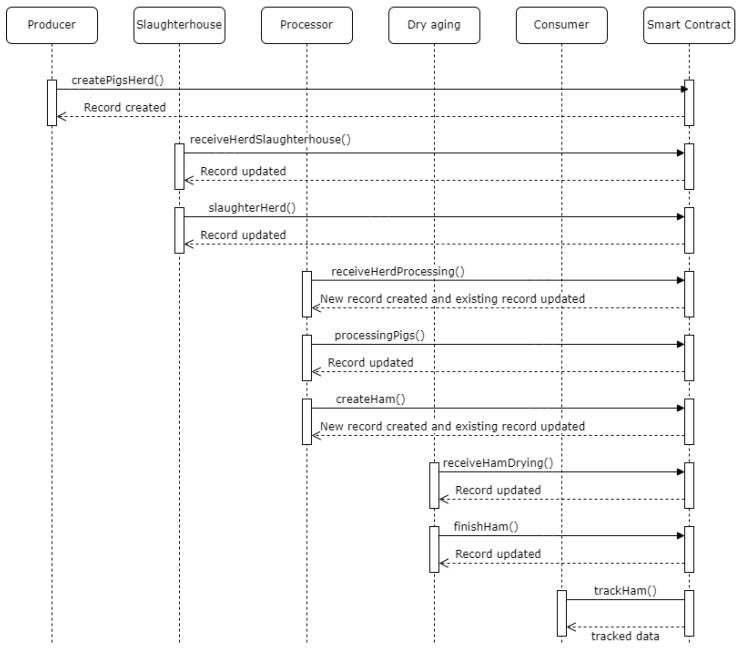
Sequence diagram representing the execution of smart contract functions throughout the product life cycle.

To better understand the developed transaction logic, Figure 9 describes the flow of execution of smart contract functions throughout the product life cycle.

### 5.3. Tests

To validate the work performed, tests were carried out on the system as a whole. To maintain the fidelity of the case study, real data were used, which were provided by the company that provided the case study.

The authors of this study were provided data from 114 hams. After careful analysis, it was concluded that they originate from three different pig herds, each from a different producer. It was also found that six different companies participated in the production cycle of these hams (three producers, one slaughterhouse, one meat processing company, and one dry aging company) so that the life cycle could be simulated according to the data provided. It was also necessary to adjust the timeline of the life cycle of the product under study (ham) to something more realistic in terms of this dissertation. This adjustment was because the timeline of the life cycle of the product in question is quite long, ranging from 1 to 3 years, which made the simulation with a real timeline unfeasible.

#### 5.3.1. Admin

The admin is the only one with permission to register new users, as well as to consult all users registered in the application.

**Figure 10:**
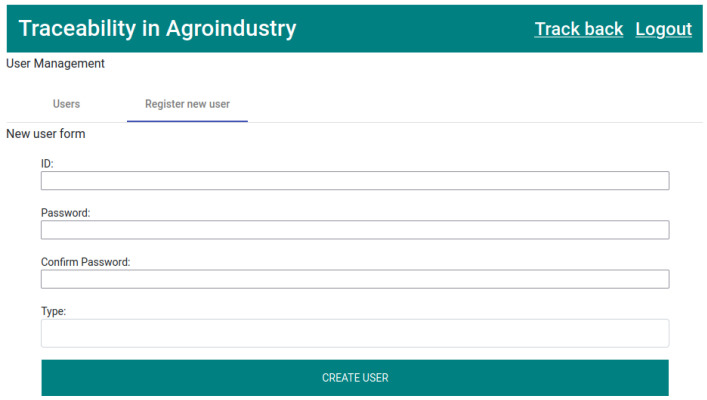
New user form.

In the application, the admin has two tabs: “Users” and “Register new user”. For the six users (three of the producer type, one of the slaughterhouse type, one of the processor type, and one of the dry aging type) to be created, the admin has to use the “Register new user” tab (Figure 10) and fill in the form for each new user.

The form has four fields, all mandatory, with these being “ID”, “Password”, “Confirm Password”, and “Type” (producer, slaughterhouse, processor, or dry aging).

**Figure 11:**
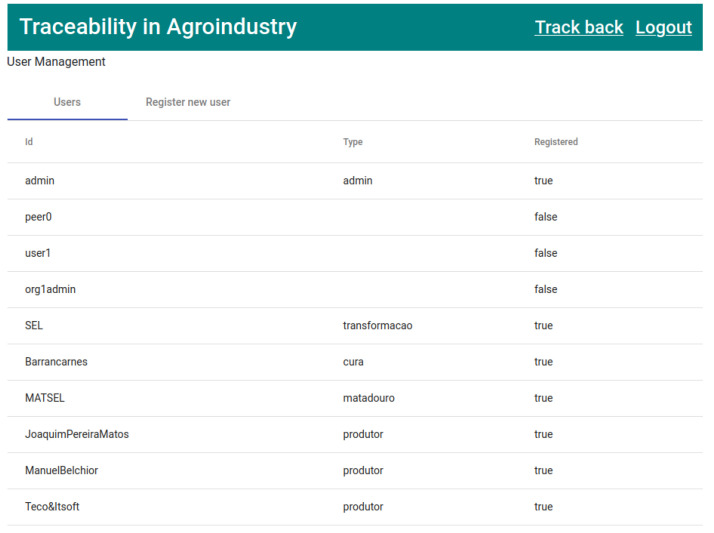
List of users registered in the system.

Once all the users were created, it was verified in the “Users” tab (Figure 11) if the identities of the previously created users were registered in the wallet. If they were (column “Registered” equals “true”), they were then allowed to access the application.

#### 5.3.2. Producer

The product life cycle, as well as the traceability process, starts at the producer with the sale of the pig herd to a processing company, which is then sent to a slaughterhouse. To represent the first state through the application, the producer must go to the “Register new pigs herd” tab and fill out the form.

**Figure 12:**
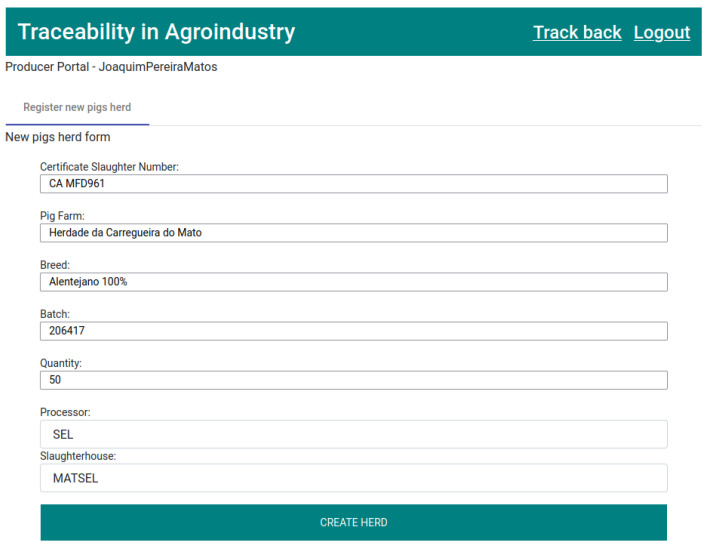
Form for creating a new pigs herd.

Filling in all the fields of the form is mandatory, with these being “Certificate Slaughter Number”, “Exploration”, “Breed”, “Batch”, “Quantity”, “Processor”, and “Slaughterhouse”, as shown in Figure 12. This process generates a new record of type, “Pigs herd”, being the key to the field “Certificate Slaughter Number”.

#### 5.3.3. Slaughterhouse

Slaughterhouse users have two tabs in their workspace, with these being “Receive herd” and “Slaughter herd”.

**Figure 13:**
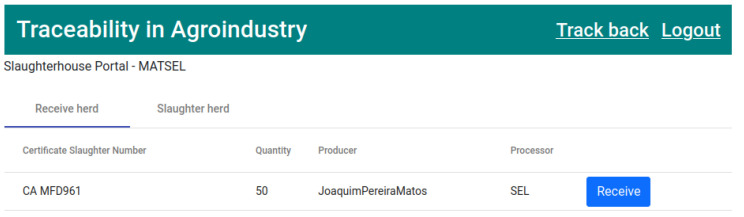
List of pig herds to be received by the slaughterhouse.

The next stage in the product life cycle is the reception of the herd at the slaughterhouse. In the “Receive herd” tab of the slaughterhouse application, the herds to be received at the premises will appear, as shown in Figure 13. The process of receiving the herd is carried out on the platform by clicking on the “Receive” button.

**Figure 14:**
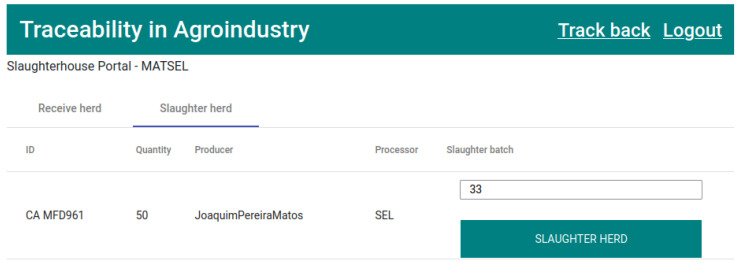
Pigs herd slaughter form.

Once the herd is received at the slaughterhouse, the next process/status is to slaughter the animals. The slaughter process requires filling in the “Slaughter batch” field on the “Slaughter herd” tab, as shown in Figure 14.

#### 5.3.4. Processor

Processor-type users have three tabs on their desktop, with these being “Receive herd”, “Processing meat”, and “Create Ham”.

**Figure 15:**
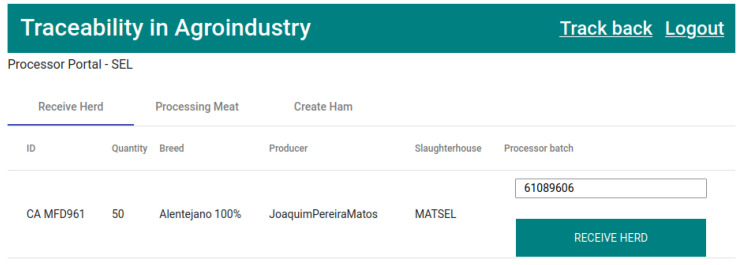
List of pig herds to be received by the processor.

The next process in the product life cycle is the reception of the herd at the meat processing company. In the “Receive Herd” tab of the meat processor to whom the pigs were sold, the herds to be received at the facility will appear, as represented in Figure 15. The transformer needs to fill in the field “Processor batch” to receive the herd. In this reception process, a new record of type “Transformation” will be created, while having “Processor batch” as the key to be entered.

**Figure 16:**
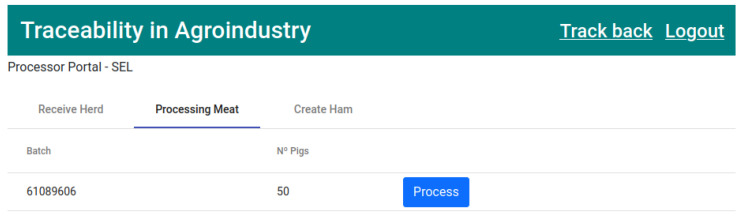
List of pig herds waiting to be transformed/processed.

Once the herd has been received by the meat processing company, the next stage of production is processing and cutting the meat. In the “Processing meat” tab, the received herds that are waiting to be cut will appear, as represented in Figure 16. This process will result in pig legs, which will finally be used to create hams.

**Figure 17:**
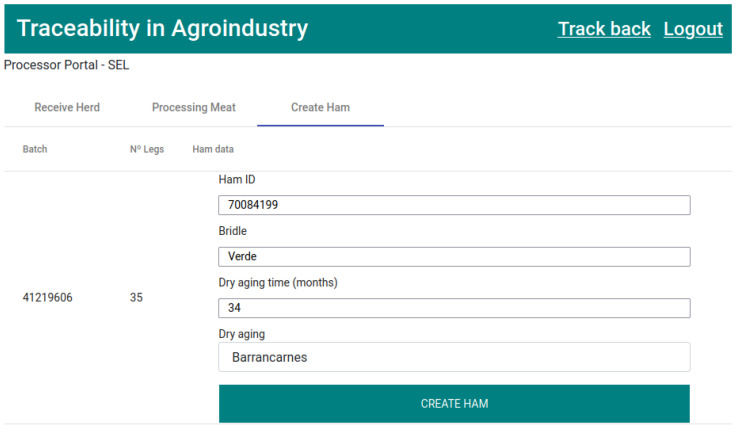
Form for creating hams.

After processing meat, in the “Create Ham” tab, the pork legs will be available for creating hams. The process of creating a ham implies the mandatory filling of the form fields: “Ham ID”, “Bridle”, “Dry aging time”, and “Dry aging” (Dry aging company), as represented in Figure 17. For each ham created, a new record of type “Hams” will be created with the entered “Ham ID” as the key.

#### 5.3.5. Dry Aging

Users of the dry aging type have two tabs in their user area, with these being “Receive Ham” and “Finish Ham”.

**Figure 18:**
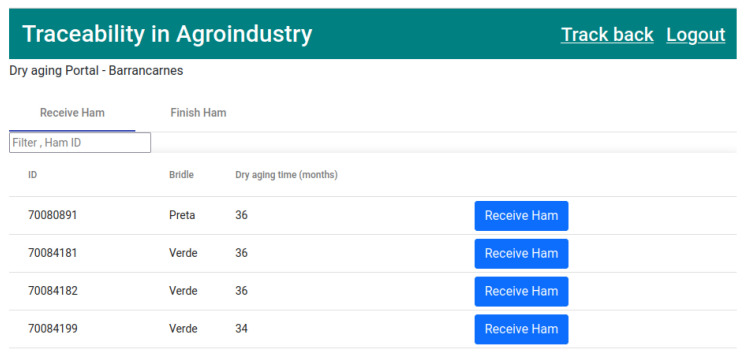
List of hams to be received by the dry aging company.

The next step in the production cycle is the receipt of the hams at the dry aging facility. In the “Receive Ham” tab of the dry aging company, which was selected by the meat processor company, the hams to be received at the facility will appear, as represented in Figure 18.

**Figure 19:**
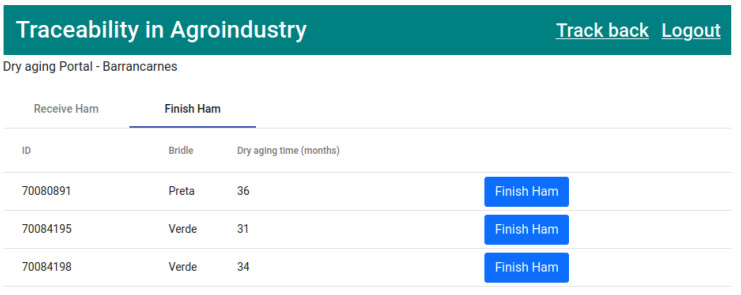
List of hams in dry aging waiting to be finished.

Finally, the last stage of the product life cycle corresponds to the finalization of the ham. The dry aging company keeps the ham on its premises for a period of time (“Dry aging time”) until the ham is cured. When the ham is ready for sale, the dry aging company can go to the “Finish Ham” tab and finish the drying process. Simultaneously, the traceability process of the ham in question is finalized, as represented in Figure 19.

#### 5.3.6. Consumer

**Figure 20:**
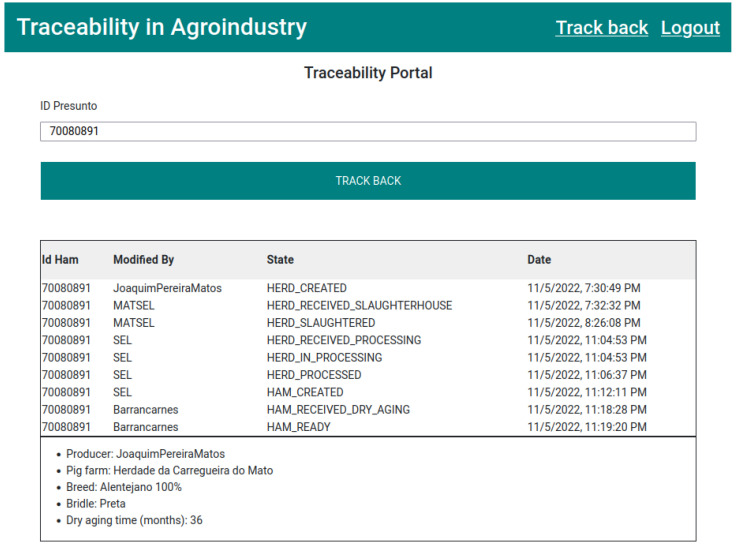
Ham information traced through the traceability portal.

Once the ham is in the consumer’s possession, the consumer can access the traceability portal and enter the identifying number that accompanies the ham in the search bar. As shown in Figure 20, the information collected throughout the production cycle will be displayed, showing its origin and the processes to which it has been subjected.

## 6. Results

This paper presents a case study, with the results section focusing on the practical use of the developed application. It provides a decentralized platform, where the actors of the product supply chain can register, update, and track all the necessary information related to each phase of the product life cycle.

As mentioned in Section 4.1, the system creates three distinct types of records along the traceability process. These three types of records are created at different instances of the product life cycle, as shown in Figure 6. Also, these records are updated over time, assuming the different states of the product; more precisely, nine states. As a result, eight transactions are generated on the blockchain, with one block for each transaction.

**Figure 21:**
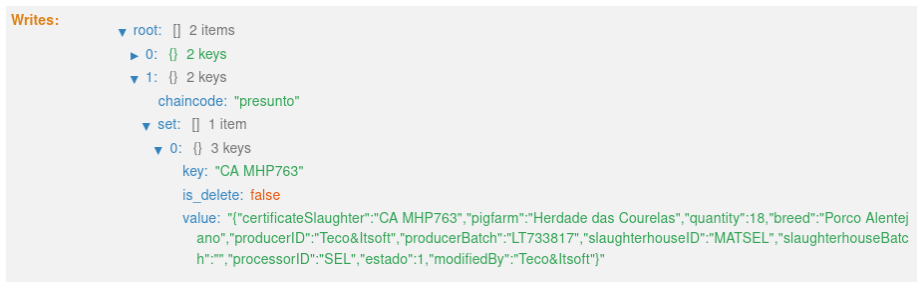
State 1.

The first transaction (Figure 21) corresponds to state 1, “HERD CREATED” (Figure 12). This executes the smart contract function *createpigsHerd()*, which corresponds to the first sequence in the diagram in Figure 9.

**Figure 22:**
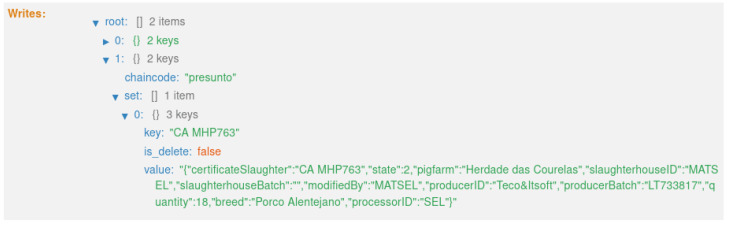
State 2.

The second transaction (Figure 22) corresponds to state 2, “HERD RECEIVED SLAUGHTERHOUSE” (Figure 13). This executes the smart contract function *receiveHerdSlaughterhouse()* (Figure 9).

**Figure 23:**
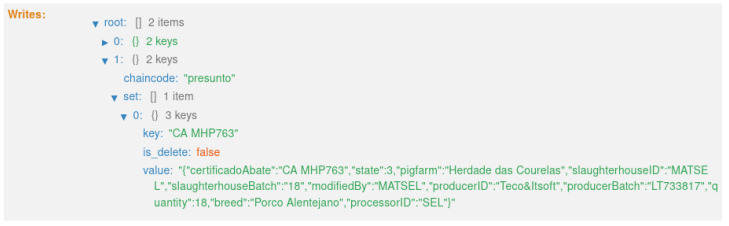
State 3.

The third transaction (Figure 23) corresponds to state 3, “HERD SLAUGHTERED” (Figure 14). This executes the smart contract function *slaughterHerd()* (Figure 9).

**Figure 24:**
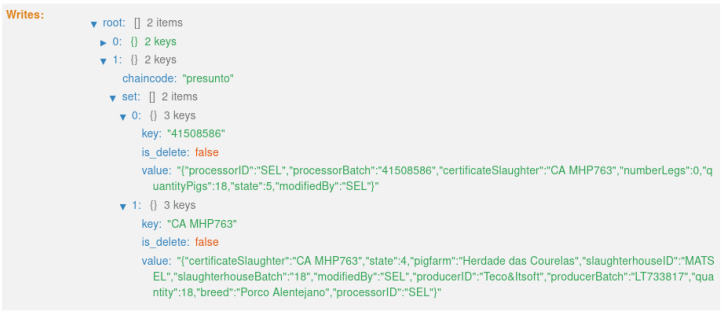
States 4 and 5.

The fourth transaction (Figure 24) corresponds to states 4 and 5,“HERD RECEIVED PROCESSING” and “HERD IN PROCESSING”, respectively (Figure 15). This executes the smart contract function *receiveHerdProcessing()* (Figure 9).

**Figure 25:**
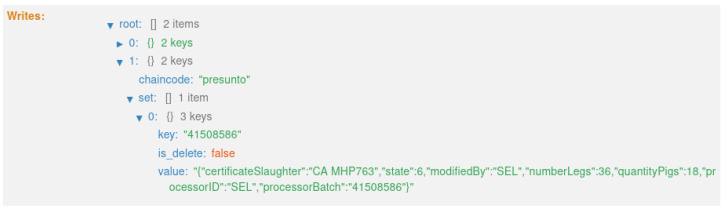
State 6.

The fifth transaction (Figure 25) corresponds to state 6, “HERD PROCESSED” (Figure 16). This executes the smart contract function *processingPigs()* (Figure 9).

**Figure 26:**
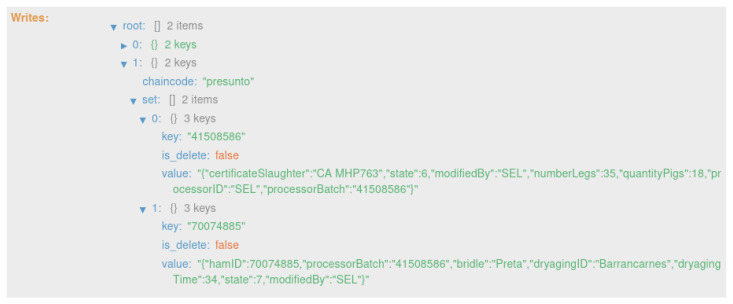
States 6 and 7.

The sixth transaction (Figure 26) corresponds to state 7, “HAM CREATED”, as well as updates state 6’s record (Figure 17). This executes the smart contract function *createHam()* (Figure 9). Once the ham is created, the number of available legs of that batch is reduced.

**Figure 27:**
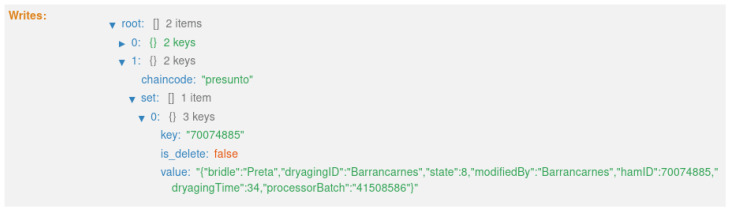
State 8.

The seventh transaction (Figure 27) corresponds to state 8 “HAM RECEIVED DRY AGING” (Figure 18). This executes the smart contract function *receiveHamDrying()* (Figure 9).

**Figure 28:**
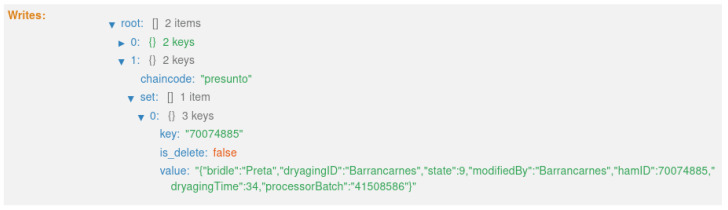
State 9.

Finally, the eighth transaction (Figure 28) corresponds to state 9, “HAM READY” (Figure 19). This executes the smart contract function *finishHam()* (Figure 9).

As can be seen from the results, the application developed allows us to obtain end-to-end traceability of the products, from the moment the pigs were sold to the completion of the ham. The data entered at the time of registration or when updating the product status is immutable and cannot be changed or deleted. This ensures their fidelity and integrity, which is essential to guaranteeing transparency of the origin and processes carried out on the product. In general, the use of the developed application allows us to demonstrate the potential of blockchain technology in conjunction with smart contracts, providing a decentralized, secure, and transparent platform. This ultimately improves and ensures the traceability and transparency of a supply chain. Therefore, it can also strengthen consumer confidence in the meat industry.

As a result, the development of the app has improved the traceability process. It has moved from a process where each entity only has access to internal process information (internal traceability), concerning where the product came from and where it is going (external traceability), to a process where everyone, including the customer, has access to all the steps the product has taken throughout its life cycle.

## 7. Discussion and Future Work

In this paper, the authors focused on the meat industry, more specifically on the production of Portuguese hams. They analyzed the requirements and challenges for implementing a blockchain-based traceability system in this type of supply chain. They developed a generalist architecture, able to be adapted to other supply chains and help anyone interested in implementing a decentralized traceability system using the HLF framework.

Despite the benefits of applying blockchain technology in food industry traceability systems, such as improved traceability and increased product transparency, there are also limitations to its application. These limitations are also valid in this research. One of the main limitations, which was mentioned in Section 5.1, is the cost of implementing and maintaining a blockchain-based application. The business paradigm in Portugal is mostly SMEs with an “against change” mentality. However, despite the high cost associated with implementing and maintaining this type of system, it can be offset thanks to the added value and competitive advantage that the end product will have over competitors. In other words, having a differentiated product can open the door to new markets or reach more demanding consumers, which can result in an increase in sales, and consequently, in production. Another limitation is the need for standardization and interoperability between different blockchain platforms to ensure that the data can be shared and integrated across different supply chains. Another limitation detected with the application of blockchain technology in traceability systems is the veracity and consistency of the data. These types of systems are dependent on the data entered by the various participants in the supply chain, which implies a risk of errors or the intentional falsification of data. This can discredit these types of systems and compromise product traceability and transparency. Another way to solve this problem is to establish protocols/rules for data entry, ensuring consistency between all participants. This process can be aided by automated tools such as sensors and/or IoT devices.

In the present work, the use of a multi-layered solution, among them blockchain, proved that it is possible to extract and store all the data necessary for the traceability of a product along the value chain, and, at the same time, ensure that the data is immutable and reliable. However, in the event that there are errors or erroneous data uncertainty on the part of elements of the supply chain, the system is prepared so that corrections can be made. All changes made are recorded on the blockchain and are visible to everyone in the chain. The development of the system using the HLF framework has significantly improved the traceability process. It allowed us to mimic the transaction logic associated with this supply chain, but also allowed for the traceability process of the hams to be carried out from the unique identification of the product. The use of the HLF framework also proved appropriate for the blockchain part. Its modular architecture allowed the real-time growth/reduction in the blockchain network, as well as the instantiation of new smart contracts. These new smart contracts could add additional data to the traceability process, be used to collect information on the supply chain (production traceability), or even represent (tokens) and digitally support current product certifications.

Future research should address the limitations mentioned above. More and more companies have been exploring blockchain technology to incorporate their traceability systems, to add value to their products. Although this work deals with the traceability of products made up of just one by-product (pork leg), this type of system also has the capacity to evolve into the traceability of products made up of several by-products, such as sausages and cheeses. However, without standardization of the data or communication between different systems, it will not be possible to achieve the full potential of blockchain technology.

## Figures and Tables

**Table 1:** Permissions by user type.

	Permissions	Create Record ^1^	Update Information ^1^	Manage Users	Track Products
Type					
Admin		X	X	X	X
Producer		X			
Slaughterhouse			X		
Processor		X	X		
Dry aging			X		
Consumer					X

^1^ When bearer of product.

**Table 2:** Product bearer in each state.

	Bearer	Producer	Slaughterhouse	Processor	Dry Aging
State					
HERD_CREATED:1		X			
HERD_RECEIVED_SLAUGHTERHOUSE:2			X		
HERD_SLAUGHTERED:3			X		
HERD_RECEIVED_PROCESSING:4				X	
HERD_IN_PROCESSING:5				X	
HERD_PROCESSED:6				X	
HAM_CREATED:7				X	
HAM_RECEIVED_DRY_AGING:8					X
HAM_READY:9					X

**Table 3:** Actions by state.

	Actions	Create Record	Update Record
State			
HERD_CREATED: 1		X	
HERD_RECEIVED_SLAUGHTERHOUSE: 2			X
HERD_SLAUGHTERED: 3			X
HERD_RECEIVED_PROCESSING: 4			X
HERD_IN_PROCESSING: 5		X	
HERD_PROCESSED: 6			X
HAM_CREATED: 7		X	X
HAM_RECEIVED_DRY_AGING:: 8			X
HAM_READY: 9			X

**Table 4:** Smart Contract Functions.

Function Name	Function Description
getCurrentUserId()	Returns the ID of the user who submitted the transaction
getCurrentUserType()	Returns the user type who submitted the transaction
createPigsHerd()	Create a new pigs herd-type record
receiveHerdSlaughterhouse()	Updates the state and information of a given pigs herd-type record.
slaughterHerd()	Updates the state and information of a given pigs herd-type record.
receiveHerdProcessing()	Updates the state and information of a given pigs herd-type record and creates a new record of type transformation
processingPigs()	Updates the state and information of a given transformation-type record.
createHam()	Create a new ham-type record and update the information of a given transformation-type record
receiveHamDrying()	Updates the state and information of a given ham-type record.
finishHam()	Updates the state and information of a given ham-type record.
queryAllRecords()	Returns all records for a given user
trackHam()	Returns all updates of all records associated with the production process of a given ham
getAllResults()	Returns all updates for a given record
batchExists()	Returns the state of a given batch

## Data Availability

The data used to support the findings of this study are the property of the company and cannot be made available to the public.

## Abbreviations

The following abbreviations are used in this manuscript:

HLF	Hyperledger Fabric
